# Surgery

**Published:** 1902-09

**Authors:** T. Carwardine


### SURGERY.

The possibility of **primary ovarian pregnancy** was for long a contested point, but several cases recorded of late have fully established the condition. It is a fact, moreover, that the ovarian pregnancy may occur as a result of impregnation through the opposite Fallopian tube. The first case on record is probably that of Bernutz and Goupil some seventy years ago, and Hastings Gilford, who described the first authentic case within recent years,[2] has analysed the literature of the subject, and concludes that sixteen cases had been previously described besides twelve other probable ones.[3]

The nature of Gilford's case was verified by Mr. Targett, although the specimen was lost when referred to the sub-committee of the Obstetrical Society. Shortly afterwards an interesting example, obtained by Prof. Kouwer, was described by Mdlle. v. Tussenbroek.[4] The patient was a healthy young mother who had sudden peritoneal hemorrhage six weeks after menstruation. The tube was perfectly normal, but the ovary presented a tumour the size of a walnut, which contained a fœtus twelve millimetres long, and a syncytium certainly developed from the fœtal ectoblast and not from the Graafian follicle. The next case was that of Croft, with the placenta in the ovary at the fourth month.[5] The ovary of the right side was replaced by a sac with a short pedicle containing a fœtus, the wall of the sac containing follicles, and the Fallopian tube patent with a normal mesosalpinx. The fœtus was of about four months' growth. Then a clear case was shown by Dr. G. P. Anning and Mr. H. Littlewood.[6] The patient was a

[1] *Lancet*, 1901, ii. [2] *Ibid.*, 1899, i. 1709.
[3] *Brit. M. J.*, 1901, ii. 964. [4] *Ann. Gynéc.*, Paris, 1899, 537.
[5] *Tr. Obst. Soc.*, 1901, xlii. 316. [6] *Ibid.*, 1902, xliii. 14.

young nullipara, who had been married five months, and was operated upon fourteen days after the last menstruation. A small ovum was found, the size of a Barcelona nut, which fitted into a firm envelope of laminated clot. This in turn fitted exactly into a recently ruptured cavity in the ovary, and the corresponding tube was normal. The specimen was presented to the Museum of the Royal College of Surgeons. Lastly, Mr. Mayo Robson has recorded a case of a young woman who was seized with sudden pain six weeks after her last menstruation.[1] A cavity was found in the ovary into which the ovum exactly fitted. From the report of Mr. Shattock, the corresponding tube was firmly adherent to the bottom of the pouch of Douglas. In the cavity of the ovary there was a recent rent three-quarters of an inch long. Into this there fitted a villous chorional sac, and a minute embryo four millimetres in length was connected with it. From the position and condition of the corresponding Fallopian tube Shattock concludes that the spermatozoa reached the ovary through the tube of the opposite side.

It will thus be seen that, since attention was specially directed to the subject, at least five authentic cases have been described within recent years. Hitherto the exigencies of the moment have prevented observation of what is probably not an uncommon condition, although formerly denied by the highest authorities.

It is now some twenty years since Hahn first performed **nephropexy.** Owing to imperfections in operative procedure, the earlier operations were very defective and were for long decried by many surgeons of note; but owing to recent improvements in technique the kidney can be securely fixed in suitable cases, and with a small mortality of something like 2 per cent. The original operations were defective chiefly from the fact that the proper capsule of the kidney alone was secured, with at most the inclusion of a small portion of the cortex. The most thorough operations of to-day include the stripping of the true capsule from a considerable portion of the kidney, or of its irritation to such an extent that it becomes covered with granulation tissue and so thoroughly incorporated with surrounding tissue during repair. As an example of the former there is the method of Edebohls referred to below, and as illustrating the latter one may refer to six cases in which I have freely applied strong carbolic acid to the surface of the kidney in conjunction with suture or gauze packing.[2]

The recent paper of Edebohls is a particularly valuable contribution to surgery, for besides its exhaustive treatment of nephropexy in general, it contributes several new ideas in technique.[3] It is hardly necessary to state that Edebohls is

[1] *Brit. M. J.*, 1902, i. 1477. [2] *Lancet*, 1902, i. 1822.

[3] *Ann. Surg.*, 1902, xxxv. 137, *et seq.*

a strong advocate for removal of the vermiform appendix when the kidney requires fixation—it is a hobby which he and a few other surgeons ride. But the vagaries of the appendix do not satisfy him, and the advantages of simultaneous exploration of the biliary region are stated as arguments for the method of operation which he proposes. He states: "In four successive nephropexies performed by the writer, lumbar exploration demonstrated the presence of gallstones in two of the patients, and of chronic cholecystitis and pericholecystitis in the other two." It is open to question, however, whether symptomless abnormalities demand surgical treatment as a rule.

The writer states that, with few exceptions, all operators by the lumbar route employ the classical incision running along the outer margin of the erector spinæ external to its sheath, separating the fibres of the latissimus dorsi, and defining the outer muscular edge of the quadratus lumborum, the remainder of the deep structures being teased through. If there is difficulty from the proximity of twelfth rib and ilium, the writer advocates the nicking of the outer margin of the quadratus lumborum near the crest of the ilium. It is then never necessary to make a trap-door incision. One conspicuous advantage of this incision is the avoidance of the ilio-hypogastric and ilio-inguinal nerves in the section, and the absence of consequent dysæsthesia.

In the preparation of the kidney for fixation, Edebohls makes it a rule to remove the fatty capsule in its entirety, and holds the view that it cannot be utilised as an aid to fixation; but he strips the true capsule from the cortex and utilises it in the fixation, passing the sutures through it and not through the parenchyma.

One of the features of the method of Edebohls is the kidney air-cushion, which presents several advantages. The lateral posture, pillows, and the hands of an assistant are thus dispensed with. It is merely an inflated india-rubber bag of suitable size placed beneath the abdomen of the patient, who is prone. The field of operation is thus rendered convex, the last rib separated from the crest of the ilium, and the kidney tends to be forced upwards into the wound. Owing to the elasticity of the pillow, respiration is easy although the patient is prone and the delivery of the kidney is largely automatic. Another principle in the writer's operation is the fixation of the kidney squarely in the loin, and so situated that the middle part of the kidney fills the lumbar space, the upper part of the kidney projecting as far upward beneath the rib as the lower pole reaches downward below the level of the crest of the ilium. This is somewhat different from the site usually adopted for fixation, and I am of opinion that it is open to the objection that the kidney in such a position is often tender.

The following is a summary of the author's procedure:—The patient is placed prone on the kidney air-cushion and a straight incision is made along the outer border of the erector spinæ. The fibres of the latissimus dorsi are then merely separated and the sheath of the erector spinæ is left unopened. The transversalis fascia is split to expose the peri-renal fat and the ilio-hypogastric nerve is drawn aside, or else temporarily divided. The sheath of the quadratus lumborum is freely opened, exposing the outer border of the muscle, and the kidney is then freed and delivered with its fatty capsule, which is then entirely removed. By an additional opening in the peritoneum the appendix can then be inspected and dealt with, as well as parts in the neighbourhood of the gall-bladder.

The next stage is the denudation of the renal cortex by nicking the true capsule, dividing it along the convexity on a director, and reflecting it forwards and backwards from the outer half of the kidney, excising a portion of the capsule if redundant. Then four suspension sutures are passed through the reflected fold of capsule in such a manner that the eversion of its edge is maintained, a Hagedorn needle being used for the purpose. The kidney is then returned and each pair of suture-ends passed through the parietes are tied together. The separated fibres of the latissimus dorsi are next united; then the skin is sutured. The dressings are left unchanged for a week. The writer adds a table of 846 operations by the best operators, giving a mortality of nearly 2 per cent. As a proof of the permanency of results, Edebohls states that the kidney so fixed in the loin can never be returned by manipulation to its normal position beneath the diaphragm; and a second exploration in one case at the patient's request showed that the kidney could not be displaced without fracturing its substance. The fact, however, that the patient had desired a second operation suggests that the results were not altogether satisfactory. Anyway, the contribution of Edebohls is a very complete and valuable one.

T. Carwardine.

